# The Effect of Individual Attitude toward Healthy Nutrition on Adherence to a High-UFA and High-Protein Diet: Results of a Randomized Controlled Trial

**DOI:** 10.3390/nu16173044

**Published:** 2024-09-09

**Authors:** Thu Huong Nguyen, Laura Pletsch-Borba, Peter H. Feindt, Caroline S. Stokes, Anne Pohrt, Nina M. T. Meyer, Charlotte Wernicke, Miriam Sommer-Ballarini, Konstantina Apostolopoulou, Silke Hornemann, Tilman Grune, Tilman Brück, Andreas F. H. Pfeiffer, Joachim Spranger, Knut Mai

**Affiliations:** 1 Department of Endocrinology and Metabolism, Charité—Universitätsmedizin Berlin, Corporate Member of Freie Universität Berlin, Humboldt-Universität zu Berlin, Charitéplatz 1, 10117 Berlin, Germany; thu-huong.nguyen@charite.de (T.H.N.);; 2 NutriAct—Competence Cluster Nutrition Research Berlin-Potsdam, 14558 Nuthetal, Germany; 3 IFST—Inclusive Food System Transition, Berlin University Alliance, 10178 Berlin, Germany; 4 BIH Charité Junior Clinician Scientist Program, BIH Biomedical Innovation Academy, Berlin Institute of Health, Charité—Universitätsmedizin Berlin, Charitéplatz 1, 10117 Berlin, Germany; 5 Albrecht Daniel Thaer—Institute of Agricultural and Horticultural Sciences, Humboldt-Universität zu Berlin, 10117 Berlin, Germany; 6 Food and Health Research Group, Faculty of Life Sciences, Albrecht Daniel Thaer—Institute of Agricultural and Horticultural Sciences, Humboldt-Universität zu Berlin, 14195 Berlin, Germany; 7 Department of Molecular Toxicology, German Institute of Human Nutrition Potsdam-Rehbrücke, 14558 Nuthetal, Germany; 8 Institute of Biometry and Clinical Epidemiology, Charité—Universitätsmedizin Berlin, Corporate Member of Freie Universität Berlin, Humboldt-Universität zu Berlin, Charitéplatz 1, 10117 Berlin, Germany; 9 Department of Human Nutrition, German Institute of Human Nutrition Potsdam-Rehbrücke, 14558 Nuthetal, Germany; 10 German Center for Diabetes Research (DZD e.V.), 85764 Neuherberg, Germany; 11 DZHK (German Centre for Cardiovascular Research), Partner Site Berlin, Berlin, Germany; 12 Leibniz Institute of Vegetable and Ornamental Crops, 14979 Großbeeren, Germany; 13 ISDC—International Security and Development Center, 10117 Berlin, Germany

**Keywords:** attitude, healthy nutrition, healthy aging

## Abstract

Despite beneficial cardiovascular effects, substantial long-term modulation of food pattern could only be achieved in a limited number of participants. The impact of attitude towards healthy nutrition (ATHN) on successful modulation of dietary behavior is unclear, especially in the elderly. We aimed to analyze whether the personal ATHN influences 12-month adherence to two different dietary intervention regimes within a 36-month randomized controlled trial. Methods: 502 subjects were randomized to an intervention group (IG; dietary pattern focused on high intake of unsaturated fatty acids (UFA), plant protein and fiber) or control group (CG; dietary recommendation in accordance with the German Society of Nutrition) within a 36-month dietary intervention trial. Sum scores for effectiveness, appreciation and practice of healthy nutrition were assessed using ATHN questionnaire during the trial (*n* = 344). Linear regression models were used to investigate associations between ATHN and dietary patterns at baseline and at month 12. Results: Retirement, higher education level, age and lower body mass index (BMI) were associated with higher ATHN sum scores. ATHN was similar in CG and IG. Higher baseline intake of polyunsaturated fatty acids (PUFA) and fiber as well as lower intake in saturated fatty acids (SFA) were associated with higher scores in practice in both groups. The intervention resulted in a stronger increase of UFA, protein and fiber in the IG after 12 months, while intake of SFA declined (*p* < 0.01). Higher scores in appreciation were significantly associated with higher intake of fiber and lower intake of SFA in the CG at month 12, whereas no associations between ATHN and macronutrient intake were observed in the IG after 12 months. Conclusions: While ATHN appeared to play a role in general dietary behavior, ATHN did not affect the success of the specific dietary intervention in the IG at month 12. Thus, the dietary intervention achieved a substantial modification of dietary pattern in the IG and was effective to override the impact of the individual ATHN on dietary behavior.

## 1. Introduction

Direct links between nutritional risk factors and non-communicable diseases including cardiovascular and metabolic diseases have been shown [1]. In recent years, many studies proposed the importance of unsaturated fatty acids (UFA) and plant protein in the prevention and management of cardiovascular diseases (CVD) and cognitive decline as well as improvement of body composition [2,3,4,5,6,7,8,9,10]. However, a huge variability of long-term adherence to dietary strategies is well known [6,11,12]. Despite the difficulty of objectively reporting adherence [13], the PREDIMED study had impressively pointed out that risk reduction in CVD is strongly associated with increment of adherence score to the Mediterranean diet [6]. Within a comparison of the effectiveness of four popular diets, a decrement of dietary adherence was seen progressively over time. Despite the ability of all four diets to reduce weight and cardiovascular risk, these effects could only be seen within participants who were highly adherent to the respective diet [11]. Risk lowering by means of higher adherence to dietary change could also be observed for stroke, cardiovascular risk and type 2 diabetes [12,14,15,16,17]. Regarding the management of type 2 diabetes, lower HbA1c and postprandial glucose levels as well as lower body mass index (BMI), waist circumference and prevalence of metabolic syndrome were associated with higher adherence to the Mediterranean diet [18]. Consequently, long-term dietary adherence is crucial in reaching and maintaining any health-related outcomes achieved through dietary changes [19,20]. To date, data that provide insight on the link between dietary adherence and its predictors is scarce and limited to small short-term studies. Known predictors of dietary adherence include socioeconomic factors (i.e., education, income, occupation), place of residence (i.e., rural, urban areas), preexisting individual dietary preferences (i.e., omnivores, vegetarians, vegans), accessibility and affordability of healthy food as well as cardiovascular risk factors (i.e., BMI, waist circumference, regular physical activity) [14,15,16,21,22,23,24]. Attitude is a psychological disposition that serves as a predictor of behavior, manifesting through positive or negative evaluations toward a specific idea or behavior [25]. Within the framework of the Theory of Planned Behavior, a positive attitude toward healthy nutrition plays a critical role in influencing an individual’s intention to adopt and maintain healthy eating habits. Besides, a strong positive attitude could help individuals navigate and overcome barriers to healthy eating, as part of the perceived behavioral control [25]. Numerous studies have demonstrated the association between attitude toward healthy nutrition and various health-related competency, including cooking skills, food skills, and dietary knowledge [26]. Furthermore, it is linked to diet quality and ability to comply with dietary guidelines across diverse participant groups [27,28,29,30]. Although attitude toward healthy nutrition (ATHN) might be relevant for the modulation of dietary behavior, data on ATHN in this context could not be found. Given its potential impact, understanding ATHN is crucial for predicting dietary behavior changes.

Previously, 12-month results of the randomized NutriAct trial showed a successful implementation of a food pattern especially focused on high intake of UFA, protein and fiber [31]. Baseline dietary pattern and participation in nutritional counseling were identified as predictors of the dietary adherence after 12 months [31]. The aim of this project was (i) to investigate ATHN in the context of numerous sociodemographic determinants, (ii) to address the relationship between individual ATHN and the macronutrient pattern at baseline and after the intervention period of 12 months. Understanding which factors are associated with ATHN and how ATHN is related to a successful modulation of dietary behavior could enable an improvement of adherence to dietary interventions, which is essential for long-term clinical benefit.

## 2. Materials and Methods

### 2.1. Study Design and Setting

Details on the NutriAct trial have been described and published previously [32]. Briefly, the NutriAct trial was designed as a randomized controlled multicenter trial in the region of Berlin-Brandenburg, Germany between 2016–2021. The main aim of this study was to assess effects of the NutriAct dietary pattern on the prevention of age-related diseases in subjects of 50 to 80 years of age with a high-risk profile for age-related diseases including cardiovascular and metabolic diseases, impaired cognitive function and/or impaired muscle strength. After initial screening, 502 subjects were included according to eligibility criteria and randomized into the intervention (IG) or control group (CG). The NutriAct pattern, which was implemented in the IG, was characterized as follows: 15 percent of total daily energy intake (%E) to 25%E protein, focusing on plant protein, UFA with 15%E to 20%E monounsaturated fatty acids (MUFA), and 10%E to 15%E polyunsaturated fatty acids (PUFA), a maximum of 10%E saturated fatty acids (SFA), 35%E to 45%E carbohydrates with low glycemic index and at least 30 g fiber per day [32]. The CG received advice following recommendations of the German Society of Nutrition (DGE) including 30%E of total fat, 55%E carbohydrates and 15%E proteins of total daily energy intake as well as 30 g fiber per day [32]. During an intervention duration of 36 months, participants were followed by means of phenotyping visits and interviews which included medical interview, physical examination, blood sampling, cognitive and physical tests at baseline (V0) and at 3 (V1), 6 (V2), 12 (V3), 24 (V4) and 36 (V5) months. Study sites of the trial included the Metabolic Research Unit of the Clinic of Endocrinology, Diabetes and Metabolism, Charité—Universitaetsmedizin Berlin and the Human Study Center of the German Institute of Human Nutrition (DIfE) Potsdam-Rehbruecke. Data collection and record were carried out using electronic case report forms (eCRF: REDCap^®^ Version Version 14.0.31). The trial protocol was approved on 08.12.2015 by the Institutional Review Board of the Charité University Medicine Berlin (EA1/315/15) and the trial was conducted according to the Declaration of Helsinki. All participants gave written informed consent. The trial was registered at German Clinical Trials Register (DRKS00010049).

### 2.2. Eligibility Criteria

Eligibility criteria were described previously [32]. In summary, male and female subjects of 50 to 80 years of age with at least one of the following risk factors for unhealthy aging were considered suitable for the NutriAct trial: known cardiovascular disease (stroke, myocardial infarction, coronary heart disease), peripheral artery disease, heart failure (according to the New York Heart Association, NYHA ≥ II or NT-Pro-BNP > 300 ng/L in absence of atrial fibrillation), elevated blood pressure (systolic blood pressure ≥ 140 mm Hg or diastolic blood pressure ≥ 90 mmHg or medical history of hypertension or use of antihypertensive medication), cognitive impairment (Montreal Cognitive Assessment, MoCA) Score < 26) or decreased physical function (Short Physical Performance Battery, SPPB Score < 10).

Exclusion criteria included acute severe CVD including unstable CVD, recent cardiovascular event, or surgery in the last 3 months. Participants were also excluded due to the usage of insulin in type 1 and type 2 diabetes, uncontrolled hypertension (blood pressure values > 180 mm Hg systolic and/or 110 mmHg diastolic), prevalent cancer, severe hepatic or renal diseases (estimated GFR < 50 mL/min/1.73 m^2^), severe gait disturbance diseases (e.g., Parkinson’s disease, stroke with paresis), severe systemic infection, severe immune disease, severe food allergy, severe malabsorption disease, oral glucocorticoid treatment, untreated active endocrine disease, severe psychiatric disorder, severe drug and/or alcohol abuse, mental limitations or known eating disorder as well as expected life expectancy less than 1 year.

### 2.3. Intervention and Control

The NutriAct dietary pattern for the IG and the dietary pattern for the CG, as outlined above, were implemented through tailored nutritional counseling sessions provided by professional dietitians. Specifically, the IG received 11 sessions, while the CG has 3 sessions during the first 12 months. Additionally, participants in the IG received rapeseed oil and specially designed food at no cost [31,32], e.g., fiber- and protein-enriched pasta, rapeseed oil and oil cake. This food supply was provided to support replacement of SFA and carbohydrate with UFA and protein during the intervention period. Food records on three consecutive days were conducted within 14 days before each visit (V0, V1, V2 and V3). Food records were converted into macronutrients according to the German federal nutrient database using PRODI^®^ (6.5 Expert; Nutri-Science GmbH, Freiburg, Germany) [32]. Intake of protein, MUFA, PUFA SFA and carbohydrate was reported as percent of total daily energy intake (%E), and fiber intake grams per day (g/d).

### 2.4. Assessment of Baseline Sociodemographic and Metabolic Characteristics

Details on data collection were previously published [32]. In brief, anthropometric measurement including weight and height was assessed at baseline using digital column scales with integrated stadiometer (Charité: Seca, Hamburg, Germany; DIfE: Soehnle, Nassau, Germany). BMI was calculated as division of weight and square of height in meters. Data of potential baseline sociodemographic predictors of dietary adherence and attitude toward healthy nutrition including sex (female/male), age, educational level (secondary school/technical course/university), occupation (retired/employed/(temporarily) unemployed), annual income (<15,000/15,000–30,000/>30,000 Euros), household (alone/shared household with at least one person) were collected using a questionnaire.

### 2.5. Attitude toward Healthy Nutrition Assessment

We assessed the participants individual attitude toward healthy nutrition with the ATHN questionnaire [33]. Effectiveness of healthy nutrition (8 questions), appreciation of healthy nutrition (8 questions), and practice of healthy nutrition (8 questions) were used to measure the individual attitude toward healthy nutrition. The fourth-dimension, consumption of low-fat food (6 questions) was not evaluated, as the investigated dietary NutriAct intervention did not focus on a low-fat diet. Participants provided answers on a 4-point scale. Sum scores for each dimension were calculated after reversed items were recoded. Higher scores indicate a stronger positive attitude toward healthy nutrition regarding each dimension. Additional details about the questionnaire are available in the supplementary materials (Supplementary Table S1). The ATHN questionnaire was carried out at V5 or after if the last visit had already occurred. Nevertheless, as all subjects were randomized at baseline, no difference in ATHN between the IG and CG would be expected at baseline. Thus, any differences in ATHN at V5 will be induced by the intervention. Out of the 502 participants, data on ATHN at 36 months and nutrition at baseline and month 12 were available for 344 participants who were included in this analysis (Supplementary Figure S1).

### 2.6. Statistical Analyses

Baseline characteristics are described as absolute counts and percentages for categorical variables and median and interquartile range for continuous variables. A total of 344 participants with available data on ATHN at 36 months and macronutrient intake at baseline and month 12 were included. To analyze differences in baseline characteristics between IG and CG analyses we caried out using Mann-Whitney U test or chi-squared test and post-hoc pairwise test, if appropriate. All analyses were caried out by means of complete case analyses.

Internal consistency of the ATHN dimensions were assessed using Cronbach’s alpha [34]. Sum scores of the ATHN dimensions are shown as median and interquartile range (IQR). Differences of the ATHN dimensions between study arms IG and CG and subgroups regarding sex, education, occupation, annual income and household size were calculated using Mann-Whitney U test for 2 groups and Kruskal-Wallis rank sum test for more than 2 groups. If there was a statistically significant difference of Kruskal-Wallis rank sum test, pairwise comparison by means of Wilcoxon rank sum test with Bonferroni adjustment was subsequently carried out. Relationships between ATHN dimensions and age as well as BMI at baseline were addressed using linear regression models.

To assess associations of the ATHN dimensions and baseline macronutrient intake in both groups (*n* = 344) we used multivariate linear models with the ATHN dimensions as independent variables and each macronutrient intake at baseline as dependent variables. Subsequently, models were adjusted for age and sex at baseline.

We conducted linear mixed models for repeated measures to assess change of each macronutrient intake within each study arm over the course of 12 months. To assess difference of macronutrient intake between the IG and CG, we used linear mixed models for repeated measures and included both groups. Here, we set time as a nested random effect within participants into the random part. As fixed effects we set study arm, time and the interaction between study arm and time. *p*-values for study arm, time (month 3, 6, 12) and the interaction between study arm and time (IG and month 3, IG and month 6, IG and month 12) were derived.

Finally, we used multivariate linear models to analyze associations between ATHN dimensions and the dietary pattern at month 12 in the IG (*n* = 170) and CG (*n* = 173, one participant was excluded due to missing data for one covariate). Subsequently, adjustments for age, sex, and BMI at baseline as well as intake of the respective macronutrient at baseline and nutritional counselling sessions were carried out. A *p*-value of < 0.05 (two-sided) showed statistical significance. All analyses were conducted using R (Version 2023.06.2).

## 3. Results

Data on ATHN and food intake were available in 344 participants (174 controls, 170 intervention subjects) of the entire study population (*n* = 502) including 128 males (37.2%) and 216 females (62.8%). The median age of this subpopulation was 66.2 years (IQR 62.9–70.4). Median baseline BMI was 28.8 kg/m^2^ (IQR 25.6–32.6). Most of the participants were retired (72.1%), had an annual income of 15,000 to 30,000 Euros (54.2%) and were educated with a technical course (81.3%). Apart from an imbalance in the distribution of annual income distribution within the middle-income group between study arms, no differences in baseline sociodemographic and baseline macronutrient intake were observed between the two groups (Table 1).

The internal consistency of ATHN dimensions with *n* = 344 using Cronbach’s alpha showed values of α = 0.823, α = 0.838, and α = 0.736, for the dimension effectiveness, appreciation and practice of healthy nutrition, respectively (Supplementary Table S2). There were no significant differences of the ATHN scores between IG and CG as well as between male and female participants (Table 2). Participants with a university degree scored higher on appreciation compared to those holding lower degrees, while retired participants scored slightly higher on practice compared to participants in employment. The highest income group (annual income ≥ 30,000 Euros) scored slightly lower on practice compared to the middle-income group (annual income between 15,000 and 30,000 Euros). In contrast, household status (single vs. shared household) did not affect any of the ATHN sum scores (Table 2). Older participants scored higher on effectiveness and on practice while participants with higher BMI scored lower on appreciation, as well as on practice (Supplementary Table S3).

**Figure 1:**
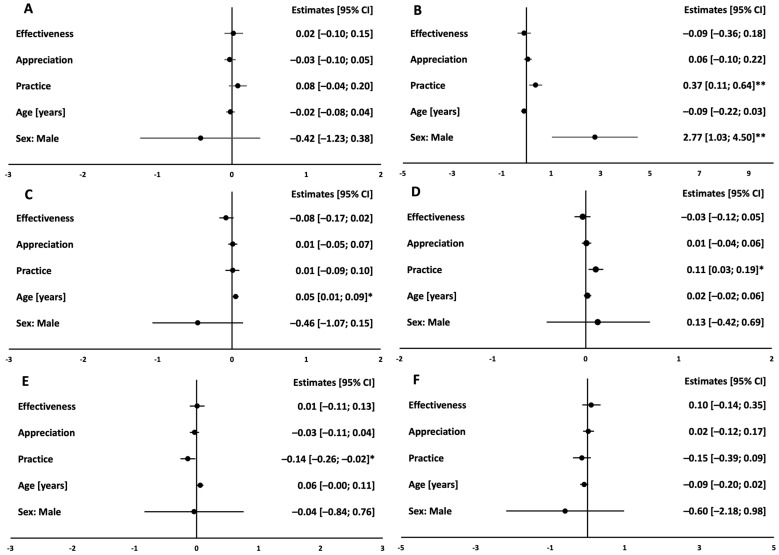
Cross-sectional associations between ATHN and baseline macronutrient pattern. Protein in %E (**A**), Fiber in g/d (**B**), MUFA in %E (**C**), PUFA in %E (**D**), SFA in %E (**E**), Carbohydrate in %E (**F**). Linear regression estimates (circles) and 95% confidence intervals (bars) of multivariate linear models showing associations of ATHN dimensions and macronutrient pattern at month 0 in both groups (*n* = 344); models were adjusted for age and sex. ** indicates significance level of *p* < 0.01, * indicates significance level of *p* < 0.05.

At baseline, all subjects with a higher score in practice had a higher baseline intake of fiber and PUFA and lower intake of SFA (all *p* < 0.05, Supplementary Table S4). These associations were confirmed after adjustment for age and sex (Figure 1).

The dietary intervention resulted in a significant increased intake of protein, fiber, MUFA and PUFA, and lower intake of SFA and carbohydrate after 12 months in the IG participants with available data on ATHN (all *p* < 0.001 vs. baseline). In the CG subjects with available ATHN data, there was a small increase in protein (*p* = 0.046), MUFA (*p* = 0.006) and PUFA (*p* < 0.001) compared to baseline, while SFA intake decreased (*p* = 0.020). No change was observed in the CG over time regarding carbohydrate and fiber intake. All changes in macronutrient intake over time were more pronounced in IG and differed significantly between IG and CG at month 12 (Supplementary Figure S2).

**Figure 2:**
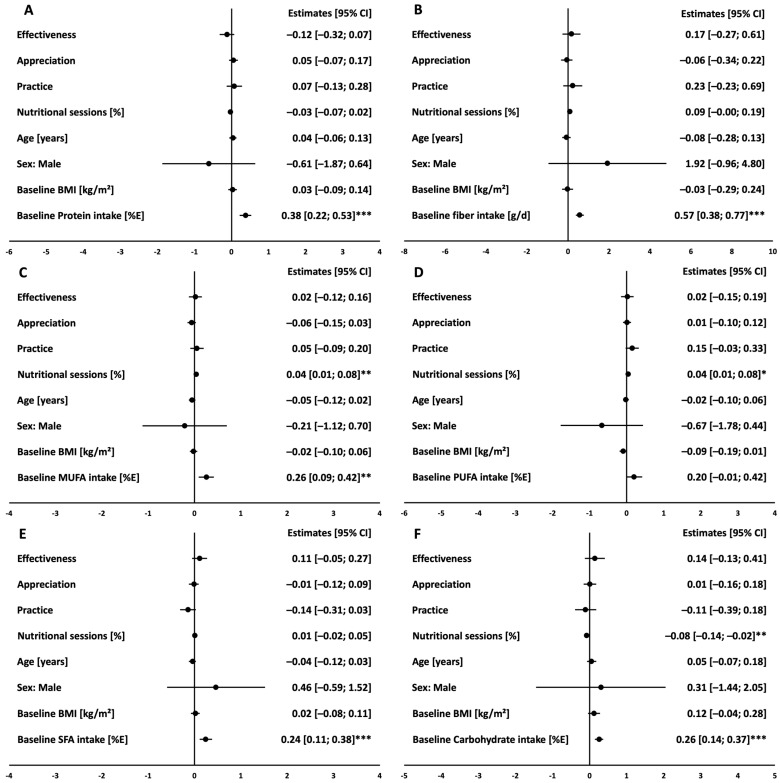
Association of the ATHN dimensions with macronutrient intake at month 12 in the IG. Protein in %E (**A**), Fiber in g/d (**B**), MUFA in %E (**C**), PUFA in %E (**D**), SFA in %E (**E**), Carbohydrate in %E (**F**). Linear regression estimates (circles) and 95% confidence intervals (bars) of multivariate linear models showing associations of ATHN dimensions and each macronutrient at month 12 in the IG (*n* = 170); models were adjusted for age, sex, BMI, nutritional counseling sessions and baseline intake of the respective macronutrient. *** indicates significance level of *p* < 0.001, ** indicates significance level of *p* < 0.01, * indicates significance level of *p* < 0.05.

Higher intake in fiber (*p* = 0.031) and PUFA (*p* = 0.002) as well as lower intake in SFA (*p* = 0.038) and carbohydrate (*p* = 0.044) was observed after 12 months in the IG participants with higher scores in practice (Supplementary Table S5). The percentage of the attended nutritional counseling sessions within the IG was associated with higher intake of MUFA and PUFA and lower carbohydrate intake at month 12 (Figure 2). Thus, the relationship between ATHN components and food intake at month 12 was further adjusted for attended nutritional sessions. After adjustment for the effect of the nutritional counseling sessions as well as the other potential confounders such as age, sex, baseline BMI, and baseline macronutrient intake of the respective macronutrient, we could not confirm an independent relationship between higher scores in practice and food intake at month 12 within the IG (Figure 2).

The abovementioned models showed that baseline intake of the respective macronutrient is associated with intake of protein, fiber, MUFA, SFA, and carbohydrate at month 12.

**Figure 3:**
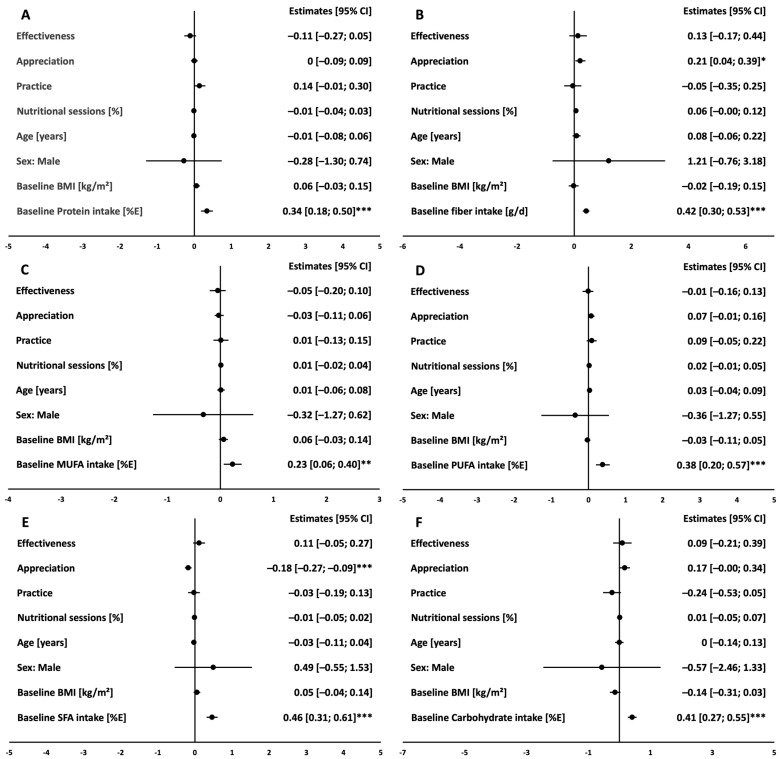
Association of the ATHN dimensions with macronutrient intake at month 12 in the CG. Protein in %E (**A**), Fiber in g/d (**B**), MUFA in %E (**C**), PUFA in %E (**D**), SFA in %E (**E**), Carbohydrate in %E (**F**). Linear regression estimates (circles) and 95% confidence intervals (bars) of multivariate linear models showing cross-sectional associations of ATHN dimensions and each macronutrient at month 12 in the CG (*n* = 173); models were adjusted for age, sex, BMI, nutritional counseling sessions and baseline intake of the respective macronutrient. *** indicates significance level of *p* < 0.001, ** indicates significance level of *p* < 0.01, * indicates significance level of *p* < 0.05.

In the CG, unadjusted linear models revealed that higher scores in appreciation were associated with higher intake in fiber (*p* = 0.006) and PUFA (*p* = 0.016) and lower intake in SFA (*p* < 0.001, Supplementary Table S6). In accordance with the findings in the IG, multivariate linear models adjusted for baseline characteristics (age, sex, BMI, baseline intake of each macronutrient) and participation on nutritional sessions showed that intake of the respective macronutrient at baseline is significantly associated with intake at month 12 in the CG (all *p* < 0.001 except for MUFA *p* = 0.008). Higher scores in appreciation remained significantly associated with higher intake of fiber and lower intake of SFA at month 12 after adjustment for the abovementioned covariates (Figure 3).

## 4. Discussion

To our best knowledge, this is the first study to assess the individual attitude of older participants towards healthy nutrition and its link to dietary adherence within a randomized dietary intervention trial in Germany.

Previously published data of the Elderly Nutrition and Health Survey in Taiwan studying participants of a similar age group suggested that nutrition attitude was more positive in men with higher education [35]. Although we could not confirm the sex effect in our cohort, our data partially supports these results regarding education as there was a positive link between higher education level and higher ATHN scores. This relationship was likely more evident in the Taiwanese study since almost 4 out of 5 participants only had primary school education or lower. The Mona Lisa-Nut study included participants in three French regions with similar educational levels compared to our study population and found an association between higher educational level and stronger attitude toward healthy eating [36], which is also in line with our results. Regarding occupational status, participants in retirement scored higher on practice compared to participants in employment. This could be attributed to the lack of time during employment as this was found to be a barrier toward healthy eating [37]. Moreover, in accordance to previous findings in elderly participants within the European NU-AGE study [38] and other cohorts [39,40], participants of our study population with higher BMI showed lower appreciation and practice scores compared to participants with lower BMI.

In general, we demonstrated that ATHN affects diet composition, as a relationship between ATHN and higher baseline intake of fiber and PUFA as well as lower SFA consumption was observed in the CG and remained significant after adjusting for potential covariates. This reflects into previous findings demonstrating a healthier diet in the context of a positive attitude toward healthy nutrition, which was independent of socioeconomic status [28]. Vice versa, Lê et al. concluded that poor attitude toward healthy nutrition in people with low socio-economic status was additionally linked to poor nutrition by means of consumption of unhealthy foods [36]. This effect seems to also persist if a low-grade dietary intervention with smaller changes regarding the dietary pattern and less frequent nutritional counseling is performed. Accordingly, ATHN has a substantial impact on the changed dietary pattern within the CG. However, this connectivity seems to be interrupted in case of strong changes in dietary pattern as intended in the IG. In fact, the relationship between intake of fiber, SFA and ATHN were no longer demonstrated in the IG after adjustment for potential confounders. Thus, it is tempting to speculate, that a counseling program including an extensive dietary modulation can somewhat override the nexus between personal ATHN and dietary behavior. This might also be partially caused by the provision of specially designed food products at no cost. Although further evidence is required to confirm this speculation, is it well known that nutritional education by means of knowledge delivery alone is not effective regarding nutritional behavior change [41]. This is likely due to the fact that determinants of food choice encompass not only physiological and neurological factors, but also psychological and environmental influences [42]. Due to the nature of our intervention, we could not address, whether other intensive dietary intervention programs can also override the link between ATHN and dietary behavior. Thus, further studies are warranted to investigate this research question.

### Strengths and Limitations

This is the first study addressing the link between the individual ATHN and two dietary interventions focusing on either the recommendations of the German Society of Nutrition or a high-UFA and high-protein diet within a dietary intervention in elderly subjects. Additionally, the study was conducted as a long-term multi-center, randomized, controlled trial with a large sample size. The provision of products including rapeseed oil, oil cake, protein-enriched pasta and cornflakes to the IG at no cost did not only facilitate the adherence to the NutriAct pattern but also mitigated financial barriers that could have otherwise hindered adherence. Within a cross-over trial comparing a Mediterranean diet with a ketogenic diet, higher adherence was equally achieved in both groups by means of providing the participants with essential food components compared to self-provision, whereas no difference regarding dietary adherence was observed between the two diet patterns [21]. Conversely, taste and financial benefits might have contributed to adherence to the NutriAct pattern, which represents a limitation of the study. Changing a dietary pattern is challenging due to the multifaceted influences of cultural, financial, social, and psychological factors, and it is not feasible to account for all these factors within one study.

Of note, we studied adherence to a diet in an older population, to whom a low-fat diet was known to be a part of a healthy diet and capable of preventing cardiovascular diseases [37,43]. Thus, generalization of our findings toward other regions or age groups should be made with caution. Moreover, since the ATHN questionnaire was conducted once at the end of the study period, we lack baseline and follow-up data. This limitation restricts our ability to study changes in ATHN over time and draw direct correlations to dietary adherence. Therefore, the assessment of long-time data with ATHN questionnaire at baseline and several follow up visits could be taken into consideration in future study designs. Nevertheless, given the randomized trial design, we initially anticipated no disparity in ATHN between the IG and CG at baseline. However, the late assessment of ATHN may have introduced a selection bias, as drop out may have been associated with ATHN scales or nutrition.

The internal consistency of the ATHN questionnaire, as indicated by Cronbach’s alpha values, suggest that the constructs measured were reliably assessed. Specifically, the high Cronbach’s alpha values for the first two dimensions, effectiveness and appreciation of healthy nutrition, indicate a strong internal consistency. The acceptable Cronbach’s alpha value of third dimension, practice of healthy nutrition, further supports the reliability of the questionnaire, although it suggests a slightly lower consistency compared to the other dimensions.

## 5. Conclusions

In the context of a nutritional intervention with less frequent nutritional counseling focusing on recommendations of the DGE as displayed in the CG, ATHN played a crucial role regarding change of macronutrient intake over time. In contrast, within an extensive dietary intervention focusing on intensive nutritional counseling in higher frequency and provision of specially designed products, change of macronutrient intake with higher percentage of daily protein and UFA intake was achieved independently of individual ATHN. According to the results of the current study, intensive intervention as designed for the IG of the NutriAct trial appears to be crucial to implement a high-UFA and high-protein diet in subjects 50 to 80 years of age to overcome the individual attitude toward healthy nutrition. Nevertheless, further studies are required to address the effects of long-term change in attitude towards nutrition on dietary adherence within different forms of diets and age groups.

## Figures and Tables

**Table 1:** Baseline sociodemographic characteristics and baseline nutritional pattern.

	Control (*n* = 174)	Intervention (*n* = 170)	Total (*n* = 344)	*p* Value
Sex [male]	65 (37.4%)	63 (37.1%)	128 (37.2%)	0.954 ^1^
Age [years]	66.5 (62.7, 71.5)	65.9 (63.2, 70.0)	66.2 (62.9, 70.4)	0.693 ^2^
BMI [kg/m^2^]	28.7 (25.6, 32.1)	29.1 (25.7, 32.7)	28.8 (25.6, 32.6)	0.590 ^2^
Education				0.843 ^1^
Secondary	7 (4.0%)	5 (3.0%)	12 (3.5%)	
Technical Course	140 (80.5%)	139 (82.2%)	279 (81.3%)	
University	27 (15.5%)	25 (14.8%)	52 (15.2%)	
N-Miss	0	1	1	
Occupation				0.574 ^1^
Retired	123 (70.7%)	123 (73.7%)	246 (72.1%)	
Employed	42 (24.1%)	39 (23.4%)	81 (23.8%)	
(Temporary) unemployed	9 (5.2%)	5 (3.0%)	14 (4.1%)	
N-Miss	0	3	3	
Annual income [Euros]				0.012 ^1^§
<15,000	30 (20.7%)	15 (10.8%)	45 (15.8%)	
15–30,000	67 (46.2%)	87 (62.6%)	154 (54.2%)	
>30,000	48 (33.1%)	37 (26.6%)	85 (29.9%)	
N-Miss	29	31	60	
Household status [shared]	124 (71.3%)	119 (70.0%)	243 (70.6%)	0.797 ^1^
Nutritional sessions [%] *	100.0 (100.0, 100.0)	90.9 (81.8, 100.0)	100.0 (81.8, 100.0)	
Protein intake [%E]	16.0 (14.4, 18.3)	15.7 (13.7, 18.4)	15.8 (13.9, 18.4)	0.336 ^2^
Fiber intake [g/d]	22.1 (17.8, 27.3)	21.8 (18.5, 27.8)	21.9 (18.0, 27.7)	0.657 ^2^
MUFA intake [%E]	13.0 (11.4, 15.2)	12.8 (11.4, 14.8)	12.9 (11.4, 15.1)	0.404 ^2^
PUFA intake [%E]	5.8 (4.5, 7.7)	5.8 (4.4, 7.1)	5.8 (4.4, 7.5)	0.814 ^2^
SFA intake [%E]	15.9 (13.5, 17.9)	15.1 (12.8, 17.7)	15.4 (13.2, 17.8)	0.109 ^2^
Carbohydrate intake [%E]	39.8 (35.0, 44.9)	41.4 (36.2, 45.4)	40.6 (35.6, 45.1)	0.141 ^2^

Data are presented as number (percentage) for categorical and median (IQR) for numerical data. ^1^ Pearson’s Chi-squared test. ^2^ Mann-Whitney U test. § Post-hoc test with p-adjustment for multiple testing shows significant difference in the middle-income group (15,000–30,000) between the IG and CG (adjusted *p* = 0.033). * is not a baseline variable, 3 and 11 nutritional counselling sessions were offered to the CG and IG during the first 12 months, respectively; the number is calculated as percentage of participated sessions to total offered sessions within each group. Abbreviation: N-Miss: number of missing data points.

**Table 2:** ATHN scores in subgroups. Data are presented as median (IQR).

	Effectiveness	Appreciation	Practice
Study arm			
Intervention (*n* = 170)	20 (18, 22)	16 (12, 20)	17 (15, 19)
Control (*n* = 174)	20 (18, 23)	15 (12, 20)	16 (14, 19)
Sex			
Female (*n* = 216)	20 (18, 22)	16 (12, 19)	17 (15, 19)
Male (*n* = 128)	20 (18, 22)	16 (11, 20)	16 (15, 19)
Education			
Secondary (*n* = 15)	20 (18, 20)	14 (9, 17) #	16 (14, 21)
Technical course (*n* = 279)	20 (18, 23)	15 (11, 19) #	17 (15, 19)
University (*n* = 52)	19 (18, 21)	19 (15, 21) #	17 (16, 19)
Occupation			
Retired (*n* = 246)	20 (18, 23)	16 (11, 20)	17 (15, 19) $
Employed (*n* = 81)	20 (18, 22)	15 (13, 19)	16 (13, 18) $
(Temporary) unemployed (*n* = 14)	18 (16, 20)	16 (8, 20)	16 (14, 18) $
Income			
<15,000 (*n* = 45)	20 (19, 23)	14 (11, 20)	16 (15, 18) §
15–30,000 (*n* = 154)	20 (18, 22)	16 (12, 20)	18 (15, 20) §
>30,000 (*n* = 85)	19 (17, 22)	16 (12, 20)	16 (14, 18) §
Household status			
Living Alone (*n* = 101)	20 (18, 23)	16 (10, 20)	17 (15, 19)
Shared Household (*n* = 243)	20 (18, 22)	16 (12, 19)	17 (15, 19)

ATHN dimensions are shown as median (IQR). Differences between subgroups were analyzed using Mann-Whitney U test for two groups and Kruskal-Wallis rank sum test for 3 groups, tests showed no significant difference unless stated otherwise. # Kruskal-Wallis rank sum test showed difference between 3 groups *p* < 0.001, subsequent pairwise comparison using Wilcoxon rank sum test with p-adjustment “Bonferroni” showed differences between university compared to secondary (*p* < 0.05) and technical course (*p* < 0.001). $ Kruskal-Wallis rank sum test showed significant difference between 3 groups *p* = 0.035, subsequent pairwise comparison using Wilcoxon rank sum test with p-adjustment “Bonferroni” showed difference between Retired and Employed (*p* = 0.049). § Kruskal-Wallis rank sum test showed significant difference between 3 groups *p* = 0.002, subsequent pairwise comparison using Wilcoxon rank sum test with p-adjustment “Bonferroni” showed difference between >30,000 and 15,000–30,000 (*p* = 0.0016).

## Data Availability

The datasets of during the current study are available from the corresponding author on reasonable request.

## Supplementary Materials

The following supporting information can be downloaded at: https://www.mdpi.com/article/10.3390/nu16173044/s1, Supplementary Table S1: Simple translation of the ATHN questionnaire; Supplementary Table S2: Cronbach’s alpha of ATHN dimensions; Supplementary Table S3: Association between age, BMI and ATHN; Supplementary Table S4: Associations between ATHN and baseline macronutrient intake; Supplementary Table S5: Associations between ATHN and macronutrient intake at month 12 in the IG; Supplementary Table S6: Associations between ATHN and macronutrient intake at month 12 in the CG; Supplementary Figure S1: CONSORT Flow diagram; Supplementary Figure S2: Changes in each macronutrient intake from baseline to month 12.
