# Supplementary material for: The Effect of Individual Attitude toward Healthy Nutrition on Adherence to a High-UFA and High-Protein Diet: Results of a Randomized Controlled Trial
Source: Nutrients. 2024 Sep 9;16(17):3044. doi: 10.3390/nu16173044 (PMC11397022; doi:10.3390/nu16173044)
Supplement: Supplementary file 1 [file nutrients-16-03044-s001.zip › nutrients-3150410-supplementary.pdf]

Supplementary materials

The effect of individual attitude toward healthy nutrition on adherence to a high-UFA and high-protein diet: results of a randomized controlled trial

Thu Huong Nguyen [1,2,3], Laura Pletsch-Borba [1,2,4], Peter H. Feindt [3,5], Caroline S. Stokes [3,6,7], Anne Pohrt [8], Nina M. T. Meyer [1,2], Charlotte Wernicke [1,2], Miriam Sommer-Ballarini [1,2], Konstantina Apostolopoulou [1], Silke Hornemann [9], Tilman Grune [2,7,10,11], Tilman Brück [3,5,12,13], Andreas F. H. Pfeiffer [1,2,9], Joachim Spranger [1,2,9,10] and Knut Mai [1,2,3,9,10,11,*]

1 Department of Endocrinology and Metabolism, Charité—Universitätsmedizin Berlin, Corporate Member of Freie Universität Berlin, Humboldt-Universität zu Berlin, Charitéplatz 1, 10117 Berlin, Germany; thu-huong.nguyen@charite.de (T.H.N.)

2 NutriAct—Competence Cluster Nutrition Research Berlin-Potsdam, 14558 Nuthetal, Germany;

3 IFST—Inclusive Food System Transition, Berlin University Alliance, 10178 Berlin, Germany;

4 BIH Charité Junior Clinician Scientist Program, BIH Biomedical Innovation Academy, Berlin Institute of Health, Charité—Universitätsmedizin Berlin, Charitéplatz 1, 10117 Berlin, Germany

5 Albrecht Daniel Thaer—Institute of Agricultural and Horticultural Sciences, Humboldt-Universität zu Berlin, 10117 Berlin, Germany

6 Food and Health Research Group, Faculty of Life Sciences, Albrecht Daniel Thaer—Institute of Agricultural and Horticultural Sciences, Humboldt-Universität zu Berlin, 10115 Berlin, Germany

7 Department of Molecular Toxicology, German Institute of Human Nutrition Potsdam-Rehbrücke, 14558 Nuthetal, Germany

8 Institute of Biometry and Clinical Epidemiology, Charité—Universitätsmedizin Berlin, Corporate Member of Freie Universität Berlin, Humboldt-Universität zu Berlin, Charitéplatz 1, 10117 Berlin, Germany;

9 Department of Human Nutrition, German Institute of Human Nutrition Potsdam-Rehbrücke, 14558 Nuthetal, Germany;

10 German Center for Diabetes Research (DZD e.V.), 85764 Neuherberg, Germany;

11 DZHK (German Centre for Cardiovascular Research), Partner Site Berlin, Berlin, Germany;

12 Leibniz Institute of Vegetable and Ornamental Crops, 14979 Großbeeren, Germany;

13 ISDC – International Security and Development Center, 10117 Berlin, Germany

* Correspondence: knut.mai@charite.de; Tel.: +49-30-450-514252, Fax: +49-30-450-514950

Supplementary Table S1: Simple translation of the ATHN questionnaire (24 items)*

Dimensions	Items (1-4 points scale)
Effectiveness of healthy nutrition	1. Nutrition research helps us to live longer.<br>2. Only by eating healthily, can we be fit and energetic.<br>3. Good nutrition is key to a long, and healthy life.<br>4. True well-being can only be reached with proper nutrition.<br>5. A healthy diet improves mental well-being also.<br>6. Many age-related issues could be avoided with a healthier diet.<br>7. Sensible dietary habits will have positive health effects for everybody.<br>8. It is certain, that one lives longer with a healthy diet.
Appreciation of healthy nutrition	1. Talking about the "right" diet has become trendy, but the issue is often taken too seriously. (reverse item)<br>2. Following every piece of advice on healthy eating can be overwhelming and confusing. (reverse item)<br>3. I am tired of hearing what food I should or should not eat. (reverse item)<br>4. If we believed everything said about nutrition, we would end up consuming nothing at all. (reverse item)<br>5. There is too much hype around nutrition. (reverse item)<br>6. It takes a long time for a healthy diet to show (if any) real effects. (reverse item)<br>7. Diet recommendations should be viewed with caution. (reverse item)<br>8. The effort to maintain a healthy diet might not be worth it, since we cannot be sure it benefits us. (reverse item)
Practice of healthy nutrition	1. I avoid consuming anything that could harm my health.<br>2. I consistently eat a healthy and balanced diet.<br>3. I place great importance that the things I eat are also healthy.<br>4. I do not worry if a snack is healthy or not. (reverse item)<br>5. I eat what I like and I do not particularly care whether it is healthy. (reverse item)<br>6. I eat what I like, and I do not pay attention to the food's contents. (reverse item)<br>7. I can confidently say that I eat healthily.<br>8. I rarely eat unhealthy food.

*Note: A back translation is necessary for use in English

Supplementary Table S2: Cronbach's alpha of ATHN dimensions

**Dimension**	**Cronbach's alpha**	**Bootstrap 95% CI based on 1000 samples [2.5%; 97.5%]**
Effectiveness	0.823	0.792; 0.850
Appreciation	0.838	0.807; 0.864
Practice	0.736	0.679; 0.784

Legend Supplementary Table S2: n=344. Each dimension contains 8 items. Cronbach's alpha was caried out after reverse items have been recoded.

Supplementary Table S3: Association between age, BMI and ATHN

	Effectiveness			Appreciation			Practice		
Predictors	*Estimates*	*CI*	*p*	*Estimates*	*CI*	*p*	*Estimates*	*CI*	*p*
Intercept	14.11	10.08 – 18.15	**<0.001**	21.33	14.82 – 27.84	**<0.001**	14.75	10.66 – 18.83	**<0.001**
Age (years)	0.08	0.03 – 0.13	**0.002**	-0.02	-0.11 – 0.06	0.618	0.08	0.02 – 0.13	**0.005**
BMI (kg/m^2)	0.00	-0.06 – 0.07	0.890	-0.16	-0.26 – -0.05	**0.003**	-0.10	-0.17 – -0.04	**0.002**
Observations	344			344			344		
R^2 / R^2 adjusted	0.027 / 0.021			0.026 / 0.020			0.052 / 0.046		

Legend Supplementary Table S3: Three linear regression models with ATHN dimensions as outcome; age and BMI were set as independent variables of each model.

Supplementary Table S4: Associations between ATHN and baseline macronutrient intake

	Protein			Fiber			MUFA			PUFA			SFA			Carbohydrate		
Predictors	*Estimates*	*CI*	*p*	*Estimates*	*CI*	*p*	*Estimates*	*CI*	*p*	*Estimates*	*CI*	*p*	*Estimates*	*CI*	*p*	*Estimates*	*CI*	*p*
Intercept	15.20	12.67 – 17.73	**<0.001**	19.26	13.73 – 24.78	**<0.001**	13.84	11.92 – 15.76	**<0.001**	4.70	2.97 – 6.43	**<0.001**	17.77	15.25 – 20.28	**<0.001**	41.12	36.15 – 46.09	**<0.001**
Effectiveness	0.02	-0.11 – 0.14	0.796	-0.11	-0.38 – 0.16	0.411	-0.06	-0.16 – 0.03	0.190	-0.03	-0.11 – 0.05	0.494	0.02	-0.10 – 0.15	0.691	0.08	-0.16 – 0.32	0.527
Appreciation	-0.02	-0.10 – 0.05	0.536	0.06	-0.10 – 0.22	0.479	0.01	-0.05 – 0.06	0.825	0.01	-0.04 – 0.06	0.800	-0.04	-0.11 – 0.04	0.311	0.04	-0.11 – 0.18	0.616
Practice	0.07	-0.05 – 0.20	0.227	0.33	0.07 – 0.60	**0.015**	0.02	-0.07 – 0.12	0.626	0.12	0.03 – 0.20	**0.007**	-0.13	-0.25 – -0.00	**0.042**	-0.17	-0.41 – 0.07	0.162
Observations	344			344			344			344			344			344		
R^2 / R^2 adjusted	0.006 / -0.003			0.023 / 0.014			0.005 / -0.004			0.024 / 0.016			0.020 / 0.011			0.006 / -0.003		

Legend Supplementary Table S4: Linear regression models with ATHN dimensions as independent variables and each macronutrient intake at baseline as independent variable of each model (n=344).

Supplementary Table S5: Associations between ATHN and macronutrient intake at month 12 in the IG

	Protein			Fiber			MUFA			PUFA			SFA			Carbohydrate		
Predictors	*Estimates*	*CI*	*p*	*Estimates*	*CI*	*p*	*Estimates*	*CI*	*p*	*Estimates*	*CI*	*p*	*Estimates*	*CI*	*p*	*Estimates*	*CI*	*p*
(Intercept)	19.35	15.18 – 23.53	**<0.001**	15.59	5.71 – 25.46	**0.002**	12.97	10.01 – 15.93	**<0.001**	5.26	1.73 – 8.78	**0.004**	13.44	10.04 – 16.85	**<0.001**	37.82	32.05 – 43.60	**<0.001**
Effectiveness	-0.12	-0.33 – 0.08	0.227	0.18	-0.30 – 0.66	0.454	-0.01	-0.16 – 0.13	0.855	0.01	-0.16 – 0.18	0.936	0.12	-0.05 – 0.28	0.159	0.19	-0.09 – 0.47	0.180
Appreciation	0.03	-0.10 – 0.16	0.604	-0.05	-0.36 – 0.25	0.737	-0.06	-0.15 – 0.03	0.216	0.01	-0.09 – 0.12	0.792	-0.02	-0.13 – 0.09	0.712	0.01	-0.17 – 0.19	0.905
Practice	0.08	-0.12 – 0.28	0.427	0.53	0.05 – 1.01	**0.031**	0.11	-0.04 – 0.25	0.143	0.27	0.09 – 0.44	**0.002**	-0.17	-0.34 – -0.01	**0.038**	-0.29	-0.57 – -0.01	**0.044**
Observations	170			170			170			170			170			170		
R^2 / R^2 adjusted	0.011 / -0.006			0.042 / 0.025			0.017 / -0.000			0.071 / 0.054			0.033 / 0.015			0.028 / 0.010		

Legend Supplementary Table S5: Linear regression models with ATHN dimensions as independent variables and each macronutrient intake at month 12 in the IG as independent variable of each model (n=170).

Supplementary Table S6: Associations between ATHN and macronutrient intake at month 12 in the CG

	Protein			Fiber			MUFA			PUFA			SFA			Carbohydrate		
Predictors	*Estimates*	*CI*	*p*	*Estimates*	*CI*	*p*	*Estimates*	*CI*	*p*	*Estimates*	*CI*	*p*	*Estimates*	*CI*	*p*	*Estimates*	*CI*	*p*
(Intercept)	17.05	13.63 – 20.47	**<0.001**	16.53	9.55 – 23.51	**<0.001**	13.99	10.99 – 16.98	**<0.001**	4.27	1.29 – 7.25	**0.005**	17.50	13.97 – 21.03	**<0.001**	39.16	32.65 – 45.66	**<0.001**
Effectiveness	-0.13	-0.30 – 0.03	0.119	0.06	-0.28 – 0.40	0.736	-0.04	-0.18 – 0.11	0.636	-0.03	-0.18 – 0.11	0.673	0.11	-0.06 – 0.28	0.207	0.12	-0.20 – 0.44	0.450
Appreciation	0.01	-0.09 – 0.10	0.915	0.28	0.08 – 0.47	**0.006**	-0.03	-0.12 – 0.05	0.435	0.10	0.02 – 0.19	**0.016**	-0.21	-0.31 – -0.11	**<0.001**	0.16	-0.02 – 0.35	0.081
Practice	0.16	-0.00 – 0.33	0.054	0.06	-0.28 – 0.40	0.728	0.00	-0.14 – 0.14	0.997	0.11	-0.03 – 0.25	0.129	-0.10	-0.27 – 0.07	0.242	-0.26	-0.57 – 0.06	0.110
Observations	174			174			174			174			174			174		
R^2 / R^2 adjusted	0.028 / 0.010			0.055 / 0.039			0.007 / -0.011			0.059 / 0.043			0.111 / 0.095			0.029 / 0.012		

Legend Supplementary Table S6: Linear regression models with ATHN dimensions as independent variables and each macronutrient intake at month 12 in the CG as independent variable of each model (n=17

Supplementary Figure S1: Consort Flow diagram

CONSORT
TRANSPARENT REPORTING of TRIALS

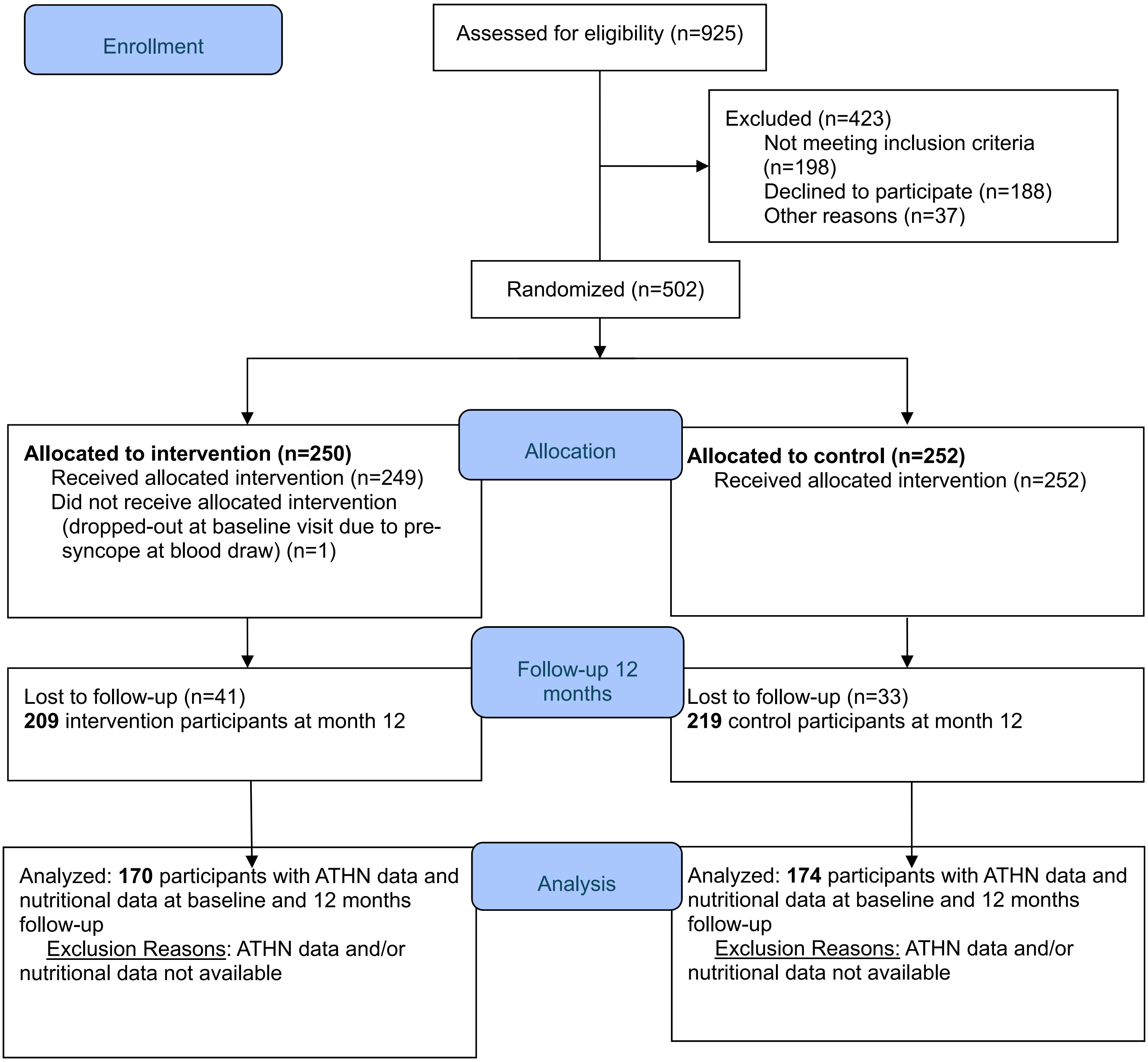

Supplementary Figure S2: Changes in each macronutrient intake from baseline to month 12

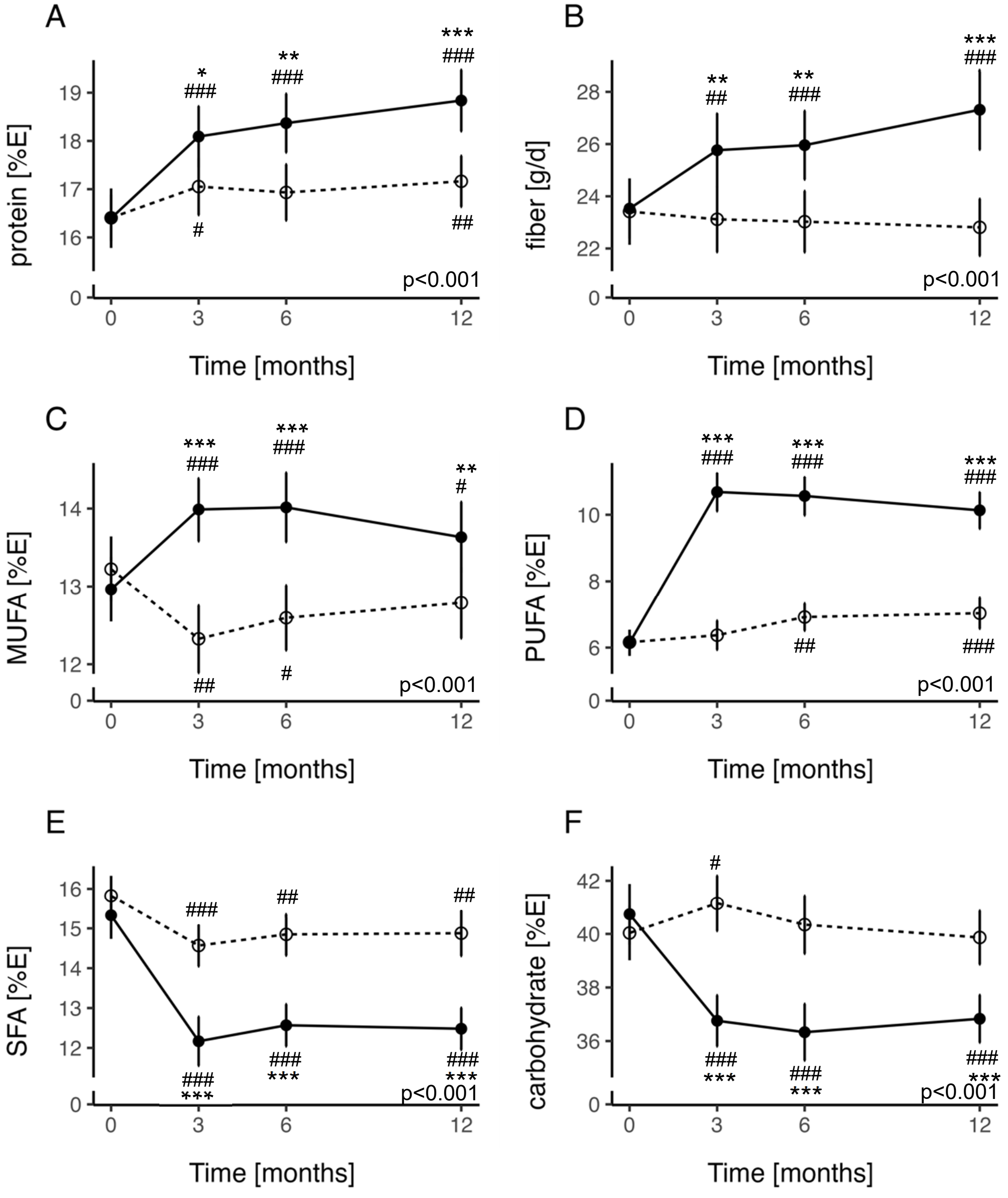

*Legend Supplementary Figure F2: Changes in macronutrient intake from baseline to month 12 in IG (black circles) and CG (white circles). Circles indicate mean and error bars show 95% confidence interval. ***, **, and * denote p-values of <0.001, <0.01 and <0.5, respectively, derived from linear mixed model for repeated measures regarding differences of macronutrient intake at each time point between IG and CG. ###, ## and # indicate p-values of <0.001, <0.01 and <0.5, respectively, regarding differences of macronutrient intake at each time point within the IG and CG compared to baseline. P-values reported for each model show significant increases in intake of the respective macronutrient over time between IG and CG.*
